# Foreign Body Ingestion: A Curious Case of the Missing Denture

**DOI:** 10.3390/geriatrics5030049

**Published:** 2020-09-13

**Authors:** Patrick Marquardt, Taylor Derousseau, Neha Patel

**Affiliations:** Department of Internal Medicine, UT Southwestern Medical Center, Dallas, TX 75390, USA; taylor.derousseau@phhs.org (T.D.); neha.patel@utsouthwestern.edu (N.P.)

**Keywords:** foreign body ingestion, denture ingestion, EGD, endoscopy

## Abstract

Foreign body ingestion is an under-recognized hazard in adults, especially in the elderly where it may lead to significant morbidity and even mortality. We present the case of an elderly patient who ingested her denture without any reported symptoms. After early recognition, endoscopic retrieval of the item was performed. We provide support for endoscopy as a safe and effective intervention for removing ingested foreign bodies in the geriatric population.

## 1. Introduction

While rates of accidental foreign body ingestion (FBI) in adults are not widely reported, a prior study estimated that 1,500 individuals in the United States die annually from this phenomenon [1]. Overall, FBI is more common in males and occurs more frequently with increasing age, especially after the seventh decade of life [2]. When ingestion occurs in adults, it is often in the setting of neurologic impairment or pathologic changes of the gastrointestinal (GI) tract [3]. In most cases, the material in question will pass through the GI tract unimpeded. However, endoscopic retrieval in adults is required in roughly 20% of cases and emergency surgery is necessary another 1% of the time [4]. We present a case of FBI in an elderly patient that resulted in a difficult, yet successful, endoscopic recovery.

## 2. Case Report

**Figure 1:**
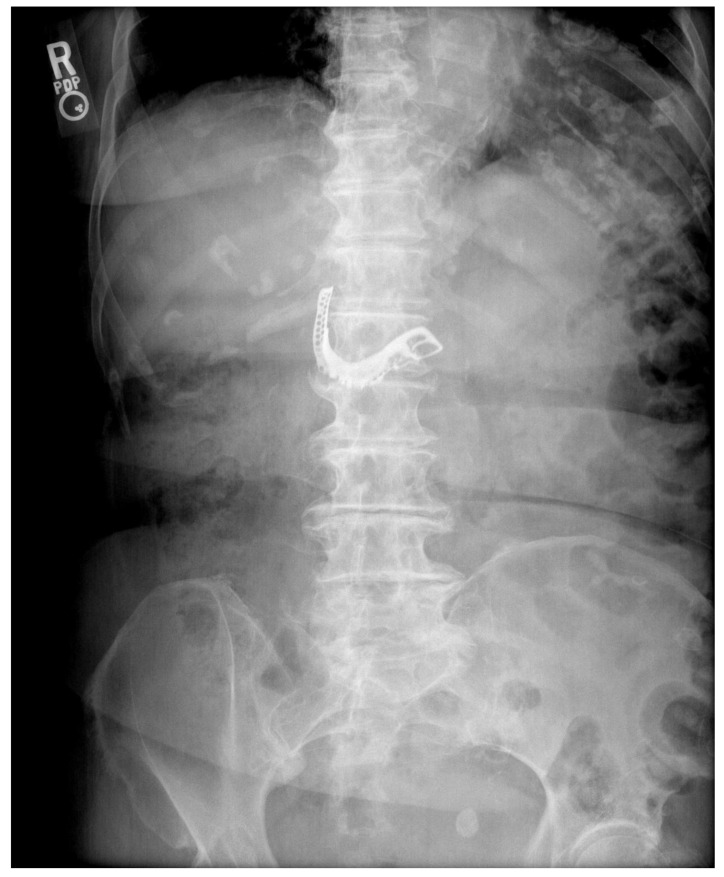
Abdominal XR.

**Figure 2:**
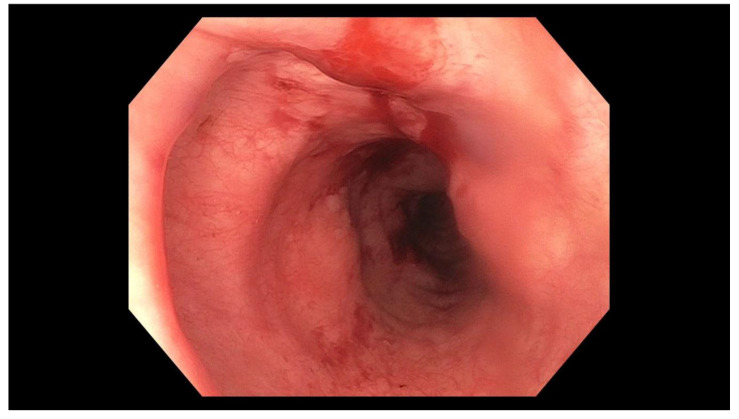
Mucosal damage in the middle third of the esophagus.

**Figure 3:**
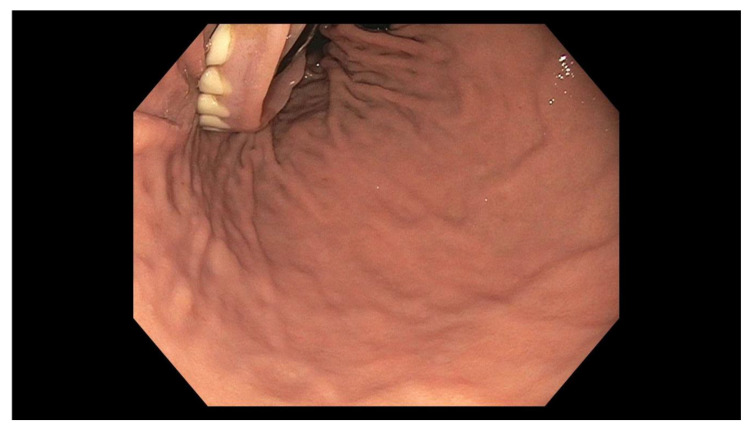
Lower denture in the gastric body on retroflexion.

The patient is an 88-year-old Asian female with a past medical history significant for Diffuse Large B-Cell Lymphoma with involvement of the brain treated with chemotherapy and radiation, Alzheimer’s dementia, and stroke. She presented to the Emergency Department due to concerns that she may have swallowed her denture. On presentation, she was unable to provide further history; however, she was accompanied by her son and daughter who did so on her behalf. They stated that the night prior, the patient’s lower denture “disappeared” while she was eating dinner. She denied experiencing any symptoms including dysphagia, dyspnea, odynophagia, abdominal pain, nausea, vomiting, hematochezia, or melena. On exam, she appeared comfortable and her vital signs were within normal limits. An acute abdominal series was obtained (Figure 1). The abdominal XR prompted an esophagogastroduodenoscopy (EGD). On first look, mucosal trauma was noted in the mid-esophagus, roughly 22–27 cm from the incisors (Figure 2). The endoscope was advanced through the lower esophageal sphincter (LES), where the intact denture was visualized in the body of the stomach (Figure 3).

**Figure 4:**
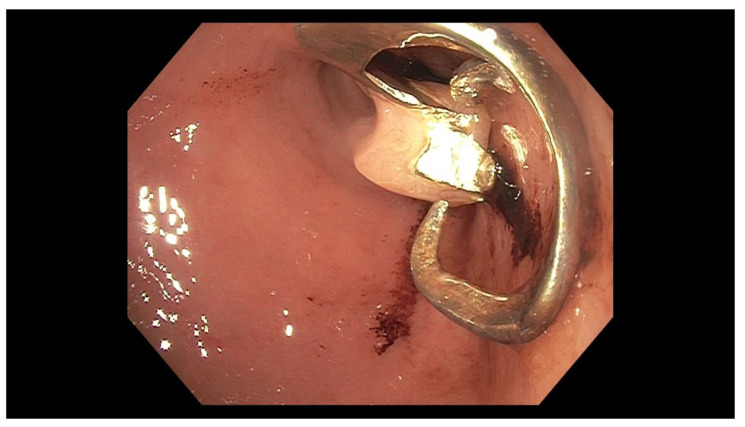
Foreign body hood protector in the mid-esophagus.

Removal of the foreign body was a technically difficult procedure. After locating the denture, the endoscope was removed and re-inserted with a foreign body hood protector attached to the tip to protect against mucosal trauma during device removal. The scope was then re-advanced, and an initial attempt was made to remove the denture. In the stomach, the hood protector was placed over the foreign body and the entire device was snared, then withdrawn to the gastroesophageal junction (GEJ). However, as it was being pulled through the GEJ, the large size of the denture and back-pressure from the sphincter caused the denture and hood protector to detach from the scope (Figure 4). Further manipulation of the device caused the snare to break, and maneuvering with rat-tooth forceps was unsuccessful. The endoscope was subsequently exchanged for another that allowed simultaneous use of snare and rat-tooth forceps. Using this combination, the endoscopist was able to grab hold of both sides of the item and safely withdraw it from the patient. On relook endoscopy, there was no significant injury related to removal of the denture. Total procedure time was 2 h and 8 min. The patient tolerated the procedure well and was able to return home the next day.

## 3. Discussion

While cases of FBI in the elderly have been presented previously, to our knowledge, the literature lacks examples of large FBI without reported symptoms. Dentures are actually one of the most commonly ingested items in adults; others include bones (chicken or fish) and jewelry [4]. Due to the large size of an item such as a denture, it often becomes impacted in the esophagus after ingestion and rarely enters the stomach. In the case of our patient, the entire denture passed through the esophagus into the stomach without any symptoms. The patient’s advanced age and neurologic spread of her lymphoma likely contributed to increased compliance and decreased contractility of her esophagus, which allowed the foreign body to pass relatively unimpeded [5]. Several factors also impacted her sensorium, including a history of dementia, stroke, and lymphoma involving the brain, making her less aware of the foreign body, especially after it had passed into the stomach. Further, a modified barium swallow study performed shortly after the episode found that the patient did not initiate a swallow with any consistency of food or drink, likely attributable to these influences. Another recognized risk factor for denture ingestion is an ill-fitting prosthetic, which individuals may inadvertently swallow during times of excitement or after trauma [6]. This may have been relevant in the case of our patient, but was not investigated. Fortunately, her caretakers noted the disappearance of the denture while she was still eating her meal. If not dealt with in a timely manner, ingested foreign bodies may cause blockage, perforation, and in some cases death [7].

This case highlights the need for thorough history taking, radiologic imaging, and possible endoscopic intervention to remove ingested items in geriatric patients. While expectant management is recommended for most cases, there are situations when endoscopy is a necessary tool to remove foreign bodies. Emergent endoscopy is warranted during episodes of esophageal obstruction, after disc battery ingestion, or after consumption of sharp or pointed objects. Non-urgent endoscopy should be considered when consumed objects measure greater than 2.5 cm or cause symptoms. Endoscopic interventions are successful in over 95% of cases of FBI, with a low likelihood of adverse events [8]. As seen in the case of our patient, dental prostheses can be some of the most difficult items to retrieve and may require advanced techniques [7,9].

In summary, FBI in older adults can be a potentially serious condition that must be dealt with expediently. Endoscopy is a safe and effective means of removing ingested foreign bodies. The patient ingested her entire denture with no apparent signs or symptoms of distress, an outcome likely related to her advanced age and comorbidities. Recognition by her caretakers led to rapid intervention, and after a difficult endoscopy, she was able to return home safely the next day.
